# Phytochemical Composition and Antioxidant Capacity of Seven Saskatoon Berry (*Amelanchier alnifolia* Nutt.) Genotypes Grown in Poland

**DOI:** 10.3390/molecules22050853

**Published:** 2017-05-21

**Authors:** Sabina Lachowicz, Jan Oszmiański, Łukasz Seliga, Stanisław Pluta

**Affiliations:** 1 Department of Fruit, Vegetables and Plant Nutraceutical Technology, Wrocław University of Environmental and Life Science, 37, Chełmońskiego Street, 51-630 Wrocław, Poland; jan.oszmianski@upwr.edu.pl (J.O.); 2 Department of Horticultural Crop Breeding, Research Institute of Horticulture, Konstytucji 3 Maja 1/3, 96-100 Skierniewice, Poland; lukasz.seliga@inhort.pl (Ł.S.); stanislaw.pluta@inhort.pl (S.P.)

**Keywords:** Saskatoon berry, polyphenolic compounds, triterpenoids, carotenoids, chlorophylls, UPLC-PDA-MS/MS

## Abstract

The basic chemical composition, bioactive compounds, and antioxidant capacity of fruits of three new Polish breeding clones (No. 5/6, type S, and type N) and four Canadian cultivars (cvs.) (“Martin”, “Smoky”, “Pembina”, and “Honeywood”) grown in Poland in 2016 were investigated. Fruits were analyzed for their contents of triterpenoids, carotenoids, chlorophylls, and polyphenolics with the ultra-performance liquid chromatography photodiode detector-quadrupole/time-of-flight mass spectrometry (UPLC-PDA-Q/TOF-MS) method, sugar with the high-performance liquid chromatography–evaporative light scattering detector (HPLC-ELSD) method, and antioxidant capacity with the ability to reduce free radical (ABTS) and ferric reducing ability of plasma (FRAP) method. Thirty-eight bioactive compounds, including twenty-eight polyphenolic compounds (four anthocyanins, nine phenolic acids, nine flavonols, and seven flavan-3-ols), four carotenoids, two chlorophylls, and three triterpenoids were identified in the fruits. The fruits of the tested Saskatoon berry genotypes were found to be rich in phenolic compounds (3773.94–6390.36 mg/100 g·dm), triterpenoids (66.55–91.31 mg/kg·dm), and carotenoids (478.62–561.57 mg/kg·dm), with high ABTS and FRAP capacity (10.38–34.49 and 9.66–25.34 mmol·Trolox/100 g·dm, respectively). Additionally, the berries of these genotypes seemed to be a good source of sugar (9.02–19.69 g/100 g), pectins (0.67%–1.33%), and ash (0.59%–0.67%). Some genotypes of Saskatoon berry, especially the clones type S, type N, and cvs. “Honeywood” and “Smoky”, may be selected for their potential applications in commercial cultivation to produce fruits with valuable health-promoting nutritional effects on human health. Additionally, three new genotypes that may offer new functional materials can be recommended for fruit growers.

## 1. Introduction

In North America, the species *Amelanchier alnifolia* Nutt., called the Saskatoon berry, has been grown and harvested commercially for many years. The fruit is a berry-like pome, red to purple to nearly black at maturity, 5–15 mm in diameter, insipid to delectably sweet, maturing at the end of June/beginning of July [1,2,3,4]. The fruits are good to eat raw, tasting somewhat like a blueberry, strongly accented by the almond-like flavor of the seeds. Saskatoon berry is a good source of typical health-promoting nutrients, and can constitute a suitable supplement for modern human nutrition. The berries contain numerous health-beneficial phytochemical compounds, mainly phenolic acids, flavonols, anthocyanins, carotenoids, minerals (manganese, calcium, potassium, magnesium, iron), vitamins (tocopherol, pyridoxine, riboflavin, ascorbic acid, riboflavin, thiamin, pantothenic acid), and pectin. In Canada, the fruits are harvested locally for pies and jams, and are very suitable for the production of various processed products. Because of the fruit’s composition, mainly concerning the content of bioactive compounds, especially polyphenols, the Saskatoon berry can play a very beneficial antioxidant, anti-inflammatory, antitumor, hypoglycemic, antidiabetic, and antiradical role, as well as regulating the level of glucose and glycogen accumulation in the human diet and has high nutritional and nutraceutical value [3,5,6,7,8,9,10]. The fruit quality and nutraceutical value of the Saskatoon berry are comparable to those of the fruit of the high-bush blueberry (*Vaccinium corymbosum* L.), but cultivation of *Amelanchier alnifolia* is much easier, because it is less demanding in terms of soil quality and weather conditions.

Poland has a long tradition as a major fruit producer and exporter of various berry crops, such as strawberry, raspberry, red and blackcurrants, gooseberry, chokeberry, and high-bush blueberry. The fruit production of most of these crops has been increasing in our country. To improve the profitability and competitiveness of small fruit production, Polish growers are interested in growing new crops, such as Saskatoon berry. The fruits of this species have unique chemical composition, taste, and usefulness both for consumers of fresh fruit and for the processing and food industry. They can be used to make drinks, jams, jellies, fruit pies, and liquors. The fruit of this crop can also be mixed with other fruits, especially those with a sour taste. Interest in the cultivation of this species is also stimulated by the natural high winter hardiness level of its plants. It means that it can be grown under the environmental conditions that exist in Poland. So far, the Saskatoon berry (*A. alnifolia)* has been cultivated on a small scale in Poland, with plans for increased fruit production in the near future [11,12].

Therefore, the aim of this study was to determine and compare polyphenols, carotenoids, chlorophylls, and triterpenoids by UPLC-PDA-Q/TOF-MS, and antioxidant capacity (the ability to reduce free radical (ABTS) and ferric reducing ability of plasma (FRAP)) in fruits of seven Saskatoon genotypes grown in a trial in Poland. In addition, the chemical properties of the fruits, such as ash, dry weight, sugar, titratable acidity, and pectin content were evaluated. Moreover, the characterization of phytochemicals and antioxidant capacity presented in this paper could offer new functional materials recommended for fruit growers and also useful for selecting the Saskatoon berry genotypes with a high content of bioactive compounds in their fruits used by the food processing industry and for the production of health beneficial products.

## 2. Results and Discussion

### 2.1. Major Chemical Compounds

The analytical results of fruit for dry weight, titratable acidity, pH, pectins, ash, and sugars of the tested Saskatoon berry genotypes are given in Table 1. Significant differences (*p* < 0.05) were revealed for the investigated basic chemical parameters among all the genotypes grown in Poland.

The average dry matter content in the fruits of the analyzed Saskatoon genotypes was 23.18 g/100 g and varied from 19.11 for clone No. 5/6 to 29.59 g/100 g for cv. “Pembina”. These results were comparable to those obtained in fruits of the Saskatoon berry cultivars grown in the Czech Republic and Canada [8,13,14]. The average value of pH of the fruits of analyzed genotypes was 3.70; the lowest value (3.02) was observed for cv. “Pembina” and the highest (3.95, 3.99) for cv. “Smoky” and clone No. 5/6, respectively. The titratable acidity and the ratio of sugar (soluble solids) to acid contents are two important determinants of fruit taste and consumer’s acceptability. The total titratable acidity in different genotypes, expressed as g/100 g of citric acid, varied from 0.37 to 0.64 g/100 g for the clones type S and No. 5/6, respectively. According to Mazza [3], the content of titratable acidity in the fruits of cv. “Smoky” was 3.12 g/100 g.

Mineral composition of the fruits depends not only on the genotypes, but also on the growing conditions [15,16]. The average content of ash in the analyzed genotypes was 0.91%, and it ranged from 0.61 to 1.14% for clone No. 5/6 and clone type S, respectively. Our results showed similar content of ash in the Saskatoon berry of tested cultivars in Canada [3]. Pectin is one form of soluble fiber responsible for the prevention of diabetes, obesity, and cardiovascular disease [17]. The average pectin amount in fruits of the tested genotypes was 0.93%; the lowest content (0.67%) was observed in cv. “Pembina” and the highest one (1.33%) in clone type S.

Fructose, sorbitol, glucose, and sucrose were the main sugars analyzed in the Saskatoon berry genotypes. The content of total sugar in the fruits of different genotypes grown in Poland ranged from 9.02 to 19.69 mg/100 g. The lowest value of this component was determined in the fruits of clone No. 5/6. The highest amount of total sugars was obtained in the fruits of another clone, type S. This content was more than double that of the lowest one observed in clone No. 5/6. The average content of total sugar in the studied genotypes was 14.78 mg/100 g, and it was 21% higher than in the Saskatoon berry genotypes evaluated in Canada [3]. In the fruits of our genotypes, fructose and glucose were the dominant sugars (31%–47% and 24%–50%, respectively; Table 1). Fruits of Canadian cv. “Martin” had the lowest glucose content (2.50 g/100 g), and clone type S had the highest one (8.76 g/100 g). The content of fructose varied from 3.14 g/100 g for clone No. 5/6 to 7.29 g/100 g for clone type S. The average fruit content of fructose, glucose, and sucrose in the analyzed genotypes was 5.47, 6.38, and 0.59 g/100 g, respectively. It was 8% lower, and 18% and 71% higher than the content of these sugars in the fruit of the Saskatoon berry genotypes analyzed by Mazza [3]. Fructose and glucose are known to be sweeter than sucrose; therefore these sugars determined the sweetness of fruits of the Saskatoon berry.

### 2.2. Identification and Quantification of Phenolic Compounds

**Figure 1:**
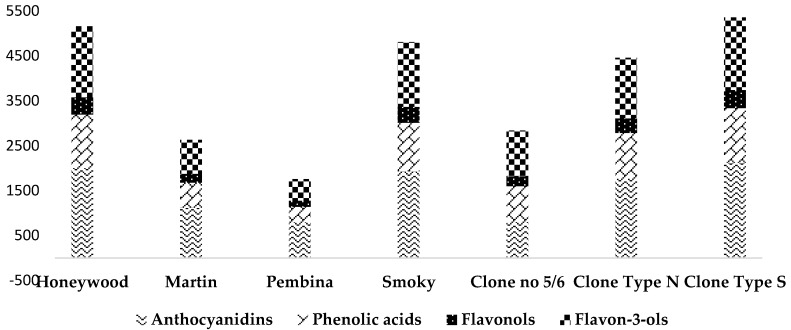
The content of polyphenolic compounds (mg/100 g·dm) of fruits in seven Saskatoon berry genotypes analyzed in 2016.

Identification and quantification of 29 compounds belonging to anthocyanins, phenolic acids, flavonols, and flavan-3-ols was based on a comparison of their retention times and ions at *m*/*z* (MS) and fragment at *m*/*z* (MS/MS) data with available standards and published data. The identification and concentration results are presented in Table 2 and Figure 1. The structures of these compounds were identified by comparison of their spectral and MS and/or MS/MS data to those reported in previous studies [9,18,19,20,21].

The polyphenolic compounds mostly contributed to the antioxidant activity of Saskatoon berries. The high content of the polyphenol antioxidants in the fruit is responsible for the observed antidiabetic, anti-inflammatory, and chemo-protective effects [10]. The concentration of the phenolic compounds, based on the UPLC-PDA-FL analysis, in Canadian cultivars (“Smokey”, “Martin”, “Pembina”, “Honeywood”) and breeding clones (No. 5/6, type N, type S) was significantly different in our studies. The average main group of phenolic compounds in fruits of the analyzed genotypes were as follows: flavan-3-ols (~46.0%) > anthocyanins (~30.0%) > phenolic acids (~18%) ≥ flavonols (~4%). The average content of phenolic compounds in the fruits of the genotypes grown in Poland was 4946.14 mg/100 g·dm. It ranged from 2992.35 to 6390.36 mg/100 g·dm for cv. “Pembina” and breeding clone type S, respectively. The highest levels of phenolic compounds (>5500 mg/100 g·dm) were found in the fruits of cvs. ”Honeywood” and ”Smoky” and breeding clones type S and type N, but the lowest content of phenolic compounds (<3500 mg/100 g·dm) was observed in clone No. 5/6 and cv. ”Pembina”. According to Lavola et al. [18], the content of phenolic compounds in fruits of cvs. ”Smoky” and ”Honeywood” grown in Finland was 50% and 38% lower than in both cultivars in our studies. However, in the study of Rop et al. [8], the fruit content of phenolic compounds in cvs. ”Smoky” and ”Martin” in the Czech Republic was 46% and 18% lower than in the same cultivars grown in Poland. The phenolic compounds’ concentration in our study was comparable to those reported in different Saskatoon berry cultivars [19,20], lower than chokeberry (*Aronia melanocarpa*) [22], and higher than cranberry (*Vaccinium macrocarpon*) fruits [23]. The phenolic compounds in fruit crops depend on many factors, such as the environmental and climatic conditions during plant growth, the place of cultivation and agricultural practices, the extraction process of fruits, the solvent, and the methodology used to identify the compounds [10,24].

#### 2.2.1. Anthocyanins

The red to purple color of the Saskatoon berry comes mainly from anthocyanins. The main fraction of polyphenols responsible for their health-promoting activities, in addition to proanthocyanidins, is anthocyanins. Anthocyanin-rich extracts have shown both anti-inflammatory effects and the induction of TNF-α [10]. The average content of total anthocyanins in the seven Saskatoon berry cultivars and clones was 1504.88 mg/100 g·dm and depended significantly on the genotype. The concentrations of anthocyanins in the fruits of analyzed genotypes ranged from 2123.43 to 798.22 mg/100 g·dm. High levels of anthocyanins (>1500 mg/100 g·dm) were found in cvs. “Honeywood” and “Smoky” and breeding clones type S and type N. The lowest level (<900 mg/100 g·dm) of anthocyanins was obtained in clone No. 5/6 and cv. ”Pembina”. According to Lavola et al. [18], the fruit content of anthocyanins in cvs. “Smoky” and “Honeywood” grown in Finland was 32% lower and 14% higher than in both cultivars in Polish conditions. In studies by Hosseinian and Beta [25], the concentration of anthocyanins was similar to our results in cv. “Smoky” and 35% higher than cv. “Martin”. The fruit content of anthocyanins of cv. “Honeywood” grown in Finland and Canada was 28% higher and 23% lower than cv. “Honeywood” grown in Poland. In our studies, the content of anthocyanins in fruits of cv. “Smoky” was 29% higher than in this cultivar grown in Canada [26]. According to Jin et al. [21], the contents of anthocyanins in cvs. “Pembina” and “Honeywood” were 50% and 31% lower than in both Saskatoon berry cultivars grown in Poland. The main anthocyanin compound in berries of seven analyzed genotypes was cyanidin-3-*O*-galactoside (57% of total anthocyanins). In the results of Lavola et al. [18] and Ozga et al. [20], cyanidin-3-*O*-galactoside was also a major compound (around 60%) of all the anthocyanins in fruits. The content of anthocyanins and their composition in the different Saskatoon berry genotypes depend on environmental factors, growing location, climatic conditions, genetic traits, cultivar, as well as on the exposure of fruits to light during the ripening of fruits [18].

#### 2.2.2. Phenolic Acids

The concentration of phenolic acids in the tested genotypes of the Saskatoon berry ranged from 564.70 to 1216.92 mg/100 g·dm; the average content was 887.64 mg/100 g·dm (Table 2). The highest levels of phenolic acids (>1100 mg/100 g·dm) were found in fruits of cv. “Honeywood” and breeding clone type S, but cvs. “Martin” and “Pembina‘’ had the lowest one (<600 mg/100 g·dm). In the fruits of all the Saskatoon genotypes grown in Poland, the chlorogenic and neochlorogenic acids were major components (45–72% and 14–22%, respectively, of the total phenolic acids). It is known that the phenolic acids, especially chlorogenic acid, have potential antioxidant activity, inhibit DNA damage, and increase the resistance of low-density lipoprotein to oxidation, and they are precursors of the flavor in fruits and vegetables [22]. According to Lavola et al. [18], chlorogenic acid was also a major compound of all the phenolic acids in different Saskatoon cultivars grown in Finland (constituting 49%, 52%, and 42% in cvs. “Smoky”, “Thiessen”, and “Honeywood”, respectively). In research by Jin et al. [21], the content of phenolic acids in the fruits of cvs. “Pembina” and “Honeywood” was 67% and 21% lower than in both cultivars analyzed in our studies.

#### 2.2.3. Flavonols

Flavonols were the smallest group of phenolic compounds found in the fruits of the Saskatoon berry (Table 2). The concentration of flavonols in fruits of the tested genotypes ranged from 141.20 to 400.91 mg/100 g·dm; the average content was 288.87 mg/100 g·dm. The lowest value of flavonols was found in the fruits of cv. “Pembina” and the highest content in the breeding clone type S. The concentrations of these compounds in the analyzed Saskatoon berry genotypes in our study were similar to those obtained in Finland and Canada [18,20]. According to Rop et al. [8], the content of flavonols in cvs. “Smoky” and “Martin” grown in the Czech Republic was 30% and 59% higher than for the same cultivars grown in Polish conditions. The main flavonol compound in berries of the seven analyzed genotypes was quercetin-3-*O*-galactoside (around 60% of the total flavonols). In the results of Lavola et al. [18], quercetin-3-*O*-galactoside was also a major compound in the fruits.

Flavonols, especially quercetin derivatives, are implicated in the berry’s health benefits, highlighted mainly for their excellent antioxidant activity. Quercetin is active in several diseases, such as cancer, neurodegenerative pathologies, and cardiovascular disease [27].

#### 2.2.4. Flavan-3-ols

Flavan-3-ols consisting of oligomers and polymeric procyanidins were the major group of the Saskatoon berry polyphenolic compounds. Flavan-3-ols are compounds that influence lipid metabolism and regulate the level of glucose and glycogen accumulation. The average content of total flavan-3-ols in the fruits of the tested genotypes was 2264.74 mg/100 g·dm. The highest (2782.24 mg/100 g·dm) concentration of total flavan-3-ols was determined in the fruits of cv. “Honeywood”, and the lowest (1395.30 mg/100 g·dm) concentration in clone No. 5/6. The major group of total flavan-3-ols in all Saskatoon genotypes grown in Poland was polymeric procyanidins (74–93%). The total content of polymeric procyanidins in analyzed genotypes ranged from 1189.76 to 2631.73 mg/100 g·dm for clone No. 5/6 and type S, respectively. The highest levels of polymeric procyanidins (>2000 mg/100 g·dm) were found in the fruits of cvs. “Honeywood” and “Smoky” and breeding clones type S and type N. The lowest (<1400 mg/100 g·dm) content of polymeric procyanidins was observed in fruits of clone No. 5/6 and cv. “Pembina”. The concentration of the monomer and dimer of flavan-3-ols in the tested genotypes of the Saskatoon berry ranged from 180 in clone type S to 444.55 mg/100 g in cv. “Pembina”. As reported by Lavola et al. [18], the varying content of flavan-3-ols in the different Saskatoon berry genotypes depends on environmental factors, climatic conditions, growing location, cultivar, and genetic traits. From the literature data, it follows that among the berries, substantial amounts of flavan-3-ols are found in chokeberries, Saskatoon berries, lingonberries, blueberries, and cranberries [10]. The flavan-3-ols concentration in our study was comparable to the values reported in different Saskatoon berry cultivars [19,20], lower than chokeberry [22], and higher than cranberry and blueberry [10,23].

### 2.3. Triterpenoid Compounds

Table 3 shows the data after identification and quantification of the triterpenoids in the fruits of seven genotypes of Saskatoon berry. The detected compounds were identified as betulinic, ursolic, and oleanolic acids based on their molecular ion [M − H]^−^ at *m*/*z* 455.3, MS profiles with the fragmentation pathways, UV-Vis spectra, and the retention times (Rt) of authentic standards. Triterpenoids have not been found in Saskatoon berries so far.

The total triterpenoid compound content in the Saskatoon berry varied depending on the evaluated genotype (Table 3). In our studies, the total triterpenoid compound content in the analyzed genotypes of the Saskatoon berry ranged from 72.21 to 91.31 mg/kg·dm for breeding clones No. 5/6 and type S, respectively. The average content of total triterpenoid compounds in the fruit of the analyzed genotypes was 79.0 mg/kg·dm. The major compound was ursolic acid (~84% of total triterpenoid compound), followed by betulinic and oleanolic acid (~12% and 5%), respectively. The total triterpenoid compound is dependent on many factors, such as climate, environmental conditions, and the degree of fruit maturity at the harvest. Triterpenoids are mainly located in cuticular waxes and exhibit many biological activities, such as antioxidant, anticancer, anti-inflammatory, and antifungal properties [28,29]. The major total triterpenoid compound in cranberry (*Vaccinium macrocarpon*) (20%), apple (*Malus domestica*) (98%), and sweet cherry (*Prunus avium*) (60% of all wax extract) was ursolic acid, as in different genotypes of the Saskatoon berry [29]. Jetter et al. [30] reported various compositions and contents of triterpenoids in the cuticular wax of many plant species. According to Szakiel et al. [31], the concentration of oleanolic (50%) and ursolic acid (28%) in bilberry (*Vaccinium myrtillus*) fruit from Finland was higher than in the Saskatoon berry. The major compounds of the total triterpenoid compound in the bilberry fruit were oleanolic and ursolic acid.

### 2.4. Composition of Carotenoids and Chlorophylls

Table 3 show the data after the identification and quantification of carotenoids and chlorophylls in seven genotypes of Saskatoon berry using UPLC-PDA-Q/TOF-MS experiments, along with their retention times, UV-Vis spectral profiles at 200–800 nm, and a comparison with standard reference compounds, when available.

Compound **1** was identified as zeaxanthin (Table 3) based on its [M + H]^+^ ion at 601.0521, which is consistent with the data reported in the literature for this compound [13]. Compound **2** was identified as all-*trans*-lutein, based on its ion at *m*/*z* 568.0021 and on the resulting product ions at *m*/*z* 551 and 553, which is consistent with the data reported in the literature for this compound [24], and with the authentic standard. Compound **3** with an identical molecular ion and fragmentation pathway was identified as 13-*cis*-lutein. This compound had absorption maxima at 335, 420, 446, and 474 nm, which is characteristic for this compound [24]. Compound **4** had molecular ions at *m*/*z* 537.1451 that fragmented at *m*/*z* 445. This compound was identified as β-carotene [13].

The total carotenoid content in the Saskatoon berry varied depending on the evaluated genotype (Table 3). The carotenoid content concentration in the analyzed genotypes ranged from 478.62 mg/kg·dm for cv. “Pembina” to 561.57 mg/kg·dm for clone type S. The average content of carotenoid content in seven Saskatoon berry genotypes grown in Poland was 514.44 mg/kg·dm. The major compounds in fruits of the Saskatoon berry were β-carotene (~68% of all carotenoid content) and lutein (~32%), followed by zeaxanthin (~0.7%). According to Mazza and Cottrell [13], the main carotenoid in fruits of cv. “Honeywood” Saskatoon berries was also β-carotene (~42% of total carotenoids), followed by lutein (~37%) and zeaxanthin (~20%).

Compound **1** was identified as chlorophyllide b due to its [M + H]^+^ ion at *m*/*z* 907.1181 and the resulting product ion at *m*/*z* 629, which is consistent with the data reported in the literature for this compound [24] and with the authentic standard. Compound 2, its [M + H]^+^ ion at *m*/z 871.1362, and the resulting product ion at *m*/*z* 593, 533, was identified as pheophytin a, in agreement with the literature data [24].

The average concentration of chlorophylls in the analyzed genotypes was 92.51 mg/kg·dm and depended significantly on the genotypes (Table 3). The content of chlorophylls in the different Saskatoon berry genotypes grown in Poland ranged from 71.2 mg/kg·dm for cv. “Smoky” to 115.15 mg/kg·dm for clone No. 5/6. It is known that the chlorophyll compounds are present mainly in the fruit and vegetable skins. In general, the fruits of all analyzed Saskatoon genotypes were characterized by low concentrations of chlorophylls. Perhaps it can be explained by the fact that the freeze-drying and analysis were conducted using fully ripe Saskatoon berries. This hypothesis was corroborated by Pumilia et al. [32], who reported that a low amount of chlorophylls is indicative of the fruit ripeness. Moreover, the green color of the fruit disappears, and the red color emerges in the ripening process of the fruits, derived mainly from anthocyanins. In addition, carotenoids are insoluble in water and occur mainly in the fruit and vegetable skin [33].

### 2.5. Antioxidant Activity and Correlations

**Figure 2:**
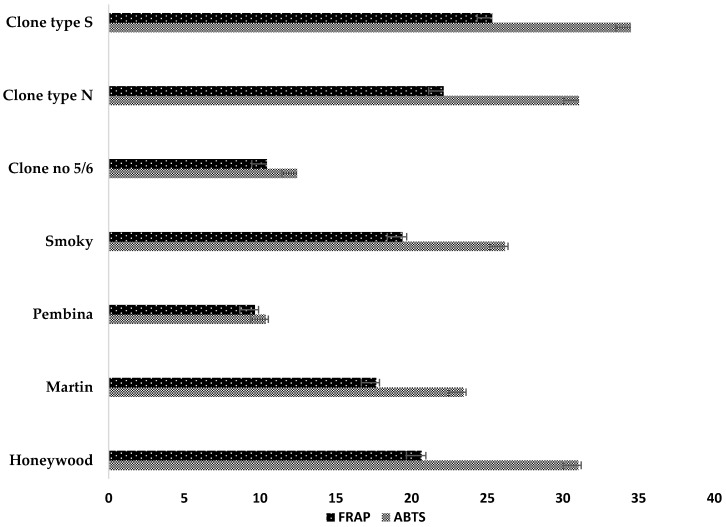
Antioxidant activity (mmol·Trolox/100 g·dm) ^1^ of different Saskatoon berry genotypes analyzed in 2016. ^1^ Values are means ± standard deviation. *n* = 3.

The results of the antioxidant capacity of the tested Saskatoon berry genotypes measured by the ABTS and FRAP methods are presented in Figure 2. Significant differences in the antioxidant capacity were observed between the analyzed genotypes using these assays. The average result of the antioxidant capacity (the ability to reduce free radical (ABTS) and ferric reducing ability of plasma (FRAP)) assay in fruits was 24.15 and 17.90 mmol·Trolox/100 g·dm, respectively. The highest antioxidant capacity (measured by both methods) in the tested genotypes was found in the Polish clone type S (34.49 and 25.34 mmol·Trolox/100 g·dm), while the lowest value was determined in the fruits of cv. “Pembina” (10.38 and 9.66 mmol·Trolox/100 g·dm). The antioxidant capacity in cv. “Smoky” grown in Poland, analyzed by the ABTS assay, was 60% lower than this cultivar grown in Canada [34]. According to Bakowska-Barczak and Kolodziejczyk [9], the antioxidant capacity analyzed by the ABTS assay was 9% lower than the value of the antioxidant capacity of the Saskatoon berry cv. “Smoky” and 9% higher than cvs. “Martin” and “Pembina”.

The correlations between the antioxidant capacity and selected phytochemicals were also examined in the studies (Table 4). The antioxidant capacity (ABTS and FRAP assay) of the analyzed genotypes of the Saskatoon berry showed the strongest correlation with the content of phenolic compounds (*r*^2^ = 0.925 for the ABTS and 0.900 for the FRAP assay). Furthermore, a significant, strong correlation was found between phenolic compounds and antioxidant capacity. Bakowska-Barczak and Kołodziejczyk [9] reported similar results for cv. “Honeywood” and stated that the antioxidant capacity correlated strongly with the anthocyanins and phenolic compounds. The type of phenolic compounds also played a very important role in antioxidant activity. Positive correlations were found between anthocyanins and polymeric procyanidinsand the results of the antioxidant capacity. Additionally, the clones type S and type N and cvs. “Honeywood” and “Smoky” high anti-radical activity was attributed to the presence of anthocyanins and polymeric procyanidins. Most carotenoids have a system of conjugated double bonds, which influence the antioxidant capacity. The antioxidant capacity of carotenoid compounds is used mainly in the scavenging of free radicals and the reduction of the risk of developing degenerative diseases. Chlorophyll has antioxidant, antimutagenic, and anticancer properties [24]. Despite the high reactivity of carotenoids and chlorophylls, positive correlations were found between carotenoid compounds, β-carotene, and lutein, and a negative correlation between chlorophylls and antioxidant capacity. Also triterpenoids, due to their antioxidative properties, exhibit anti-HIV activity and showed significant cytotoxicity in leukemia cells as well as anticancer, antioxidative, and anti-inflammatory properties, and a significant positive correlation was found between triterpenoid compounds and the antioxidant capacity [28,29]. In the studies of Loza-Mejía and Salazar [28] and Szakiel et al. [29], the antioxidant capacity of the fruit was found to be strongly and positively correlated with the triterpenoids compound (*p* = 0.05, *r*^2^ = 0.621 for ABTS and *r*^2^ = 0.607 for FRAP). Therefore, it could be concluded that the antioxidant capacity of the tested Saskatoon genotypes correlated with the levels of the phenolic compounds, anthocyanins, flavonols, procyanidins polymeric, carotenoid compounds, and triterpenoid compounds. Moreover, the antioxidant capacity of the Saskatoon berry depended on the chemical composition of the antioxidant compounds [8].

### 2.6. Principal Component Analysis (PCA)

**Figure 3:**
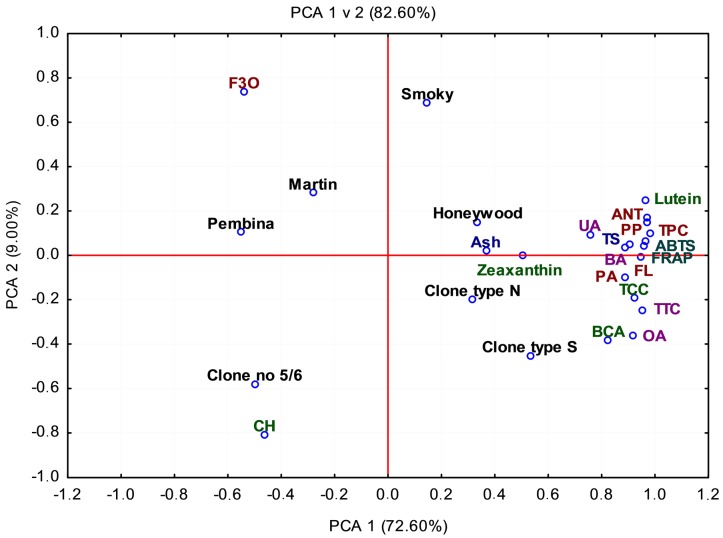
PCA mean showing the relationship among phytochemical compounds and antioxidant activity in fruits of the Saskatoon genotypes grown in Poland. UA, ursolic acid; OA, oleanolic acid; BA, betulinic acid; FL, flavonols; ANT, anthocyanins; PA, phenolic acid; F3O, falavan-3-ols; BCN, beta-carotene; CH, chlorophylls; TS, total sugar; TCC, total carotenoids compounds; TTC, total triterpenoids compounds; PP, polymeric procyanidins.

The average results of our studies obtained for seven different genotypes (three Polish clones No. 5/6, type S, and type N, and four Canadian cultivars “Martin”, “Smoky”, “Pembina”, and “Honeywood”) of the Saskatoon berry in their bioactive composition and the antioxidant activity using principal component analysis (PCA) are presented in Figure 3. Two main PCAs for the analysis of the seven genotypes grown in Poland accounted for 82.60% of the total variability, PC1 for 71.60% and PC2 for 9.00% (Figure 3). The results obtained from PCA using the linkage method among the groups indicated the presence of four clusters:

(1) clones type S and type N with the highest concentrations of oleanolic acid (OA), flavonols (FL), phenolic acids (PA), total carotenoid compounds (TCC), β-carotene (BCA), total triterpenoid compounds (TTC), and zeaxanthin. Additionally, a positive correlation with antioxidant capacity was detected.
(2) cvs. “Smoky” and “Honeywood” with the high antioxidant activity (ABTS, FRAP), and high contents of anthocyanins (ANT), phenolic compounds (PC), betulinic acid (BA), ursolic acid (UA), polymeric procyanidin (PP), total sugar (TS), ash, and lutein. Additionally, a positive correlation with antioxidant capacity was detected.
(3) cvs. “Martin” and “Pembina” with high contents of flavan-3-ols (F3o) and a positive correlation with antioxidant capacity.
(4) clone No. 5/6 with the highest content of chlorophylls (CH) and low antioxidant capacity.

## 3. Materials and Methods

### 3.1. Reagent and Standard

Acetonitrile, formic acid, methanol, all-*trans*-β-carotene, α-carotene, all-*trans*-lutein, neoxanthin, violaxanthin, antheraxanthin, β-cryptoxanthin, chlorophyll a, betulinic, oleanolic and ursolic acid, ABTS (2,2′-azinobis(3-ethylbenzothiazoline-6-sulfonic acid), 6-hydroxy-2,5,7,8-tetramethylchroman-2-carboxylic acid (Trolox), 2,4,6-tri(2-pyridyl)-s-triazine (TPTZ), methanol acetic acid, and phloroglucinol were purchased from Sigma-Aldrich (Steinheim, Germany). (−)-Epicatechin, (+)-catechin, procyanidin A2 and B2 chlorogenic acid, neochlorogenic acid, cryptochlorogenic acid, di-caffeic quinic acid, p-coumaric acid, 4-caffeoylquinic, kampferol-3-galactoside, quercetin-3-*O*-rutinoside, quercetin-3-*O*-galactoside, quercetin-3-*O*-glucoside, quercetin-3-*O*-arabinoside, quercetin-3-*O*-xyloside, quercetin-deoxyhexo-hexoside caffeic acid, cyanidin-3-*O*-galactoside, cyanidin-3-*O*-glucoside, cyanidin-3-*O*-arabinoside, and cyanidin-3-*O*-xyloside were purchased from Extrasynthese (Lyon, France). Acetonitrile for ultra-phase liquid chromatography (UPLC; Gradient grade) and ascorbic acid were purchased from Merck (Darmstadt, Germany).

### 3.2. Plant Materials

Fruits of the Saskatoon berry (*A. alnifolia*) of seven genotypes (three Polish clones No. 5/6, type S, and type N, and four Canadian cultivars “Martin”, “Smoky”, “Pembina”, and “Honeywood”) were used in the study. Fruit samples (~1.0 kg each) of six genotypes were collected from bushes grown in a field, belonging to the Research Institute of Horticulture in Skierniewice, Central Poland (51°55′24″ N, 020°5′58″ E). The one fruit sample of Canadian cultivar “Honeywood” was collected from the horticultural farm in Wojciechów near Lublin, Poland (51°14′08″ N 22°14′41″ E). The plant material for the field experiment was derived from vegetative propagation in an in vitro culture in a tissue culture laboratory at the Research Institute of Horticulture in Skierniewice. Four Canadian cultivars of the Saskatoon berry (*Amelanchier alnifolia* Nutt.), such as “Martin”, “Smoky”, “Pembina”, and “Honeywood” were imported from Canada’s tissue culture laboratory in 2007 as multiply (“Multiplat”) potted plants (“72 Cell Liner plugs”). Three Polish breeding clones No. 5/6, type S, and type N were selected by Prof. Stanisław Pluta in 2007/2008 at the Plant Breeding Department of the Horticultural Institute. In 2010, all genotypes (4 cultivars and 3 clones) were multiplied in in vitro cultures in the above-mentioned tissue culture laboratory at the Institute of Horticulture in Skierniewice. Propagated and planted plant material was used to establish the field trial in 2011 in the Experimental Orchard at Dąbrowice.

Fruits were collected at the optimum ripening time in 2016. The fresh fruits were directly frozen at −25°C, stored at −20°C, and then freeze-dried (24 h; Christ Alpha 1–4 LSC; Osterode am Harz, Germany). The homogeneous dry material was obtained by crushing the dried tissues using a closed laboratory mill (IKA A.11, Werke Staufen, Breisgau, Germany). The powders were kept in a refrigerator (−80 °C) until extract preparation.

### 3.3. Identification and Quantification of Polyphenols

Qualitative (LC/MS QTOF) and quantitative (UPLC-PDA-FL) analysis of polyphenols (anthocyanin, flavan-3-ol, flavonol, and phenolic acid) was performed as described previously by Oszmiański and Lachowicz [22]. All measurements were repeated three times. The results were expressed as mg per 100 grams of dry matter (dm).

### 3.4. Analysis of Proanthocyanidins by the Phloroglucinolysis Method

Direct phloroglucinolysis of freeze-dried samples was performed as described by Oszmiański et al. [35] using reverse-phase high-performance liquid chromatography (RP-HPLC) analysis, and phloroglucinol products were separated on a Cadenza CD C18 (75–4.6 mm, 3 μm) column (Imtakt, Kyoto, Japan) All data were obtained in triplicate. The results were expressed as mg per 100 g·dm.

### 3.5. Identification and Quantification of Carotenoids and Chlorophylls

For the extraction of carotenoids, a protocol similar to that described previously was applied [36]. Samples (10 μL) were eluted according to the linear gradient described by Delphino-Rius et al. [37]. The runs were monitored at 450 and 650 nm. The PDA spectra were measured over the wavelength range of 200–700 nm in steps of 2 nm. The retention times and spectra were compared to those of the authentic standards. All incubations were done in triplicate. The results were expressed as mg per kg of dm.

### 3.6. Identification and Quantification of Triterpenoids

Fruit sample extraction was performed as described by Farneti et al. [38]. Identification and quantification of ursolic, oleanolic, and betulinic acids was done using the ACQUITY Ultra Performance LC system with a binary solvent manager (Waters Corp., Milford, MA, USA), a UPLC BEH C18 column (1.7 μm, 2.1 mm × 150 mm, Waters Corp., Milford, MA, USA), and a Q-TOF mass spectrometer (Waters, Manchester, UK) equipped with an electrospray ionization (ESI) source, operating in negative mode. The elution solvent was methanol–acetonitrile (15:85, *v*/*v*), at a flow rate of 0.1 mL·min^−1^. The *m*/*z* for betulinic acid was 455.3452, for oleanolic acid 455.3496, and for ursolic acid 455.3365, and the retention times were 6.89, 7.58, and 8.19 min, respectively. The compounds were monitored at 210 nm. All data were obtained in triplicate. The results were expressed as mg per kg of dm.

### 3.7. Determination of Antioxidant Activity

The solvent for the analysis was prepared as described previously by Lachowicz et al. [39]. The ABTS and the FRAP assays were determined according to Re et al. [40] and Benzie and Strain [41]. Determinations by the ABTS and FRAP methods were performed using the UV-2401 PC spectrophotometer (Shimadzu, Kyoto, Japan). The antioxidant activity was expressed as mmol of Trolox per 100 g·dm.

### 3.8. Analysis of Sugar by the HPLC-ELSD Method

An analysis of sugar by the HPLC-ELSD method was performed according to the protocol described by Oszmiański and Lachowicz [22]. Calibration curves (*R*^2^ = 0.9999) were created for glucose, fructose, sorbitol, and sucrose. All data were obtained in triplicate. The results were expressed as mg per 100 g·dm.

### 3.9. Statistical Analysis

Statistical analysis, one-way ANOVA and principal component analysis (PCA) were conducted using Statistica version 12.5 (StatSoft, Kraków, Poland). Significant differences (*p* ≤ 0.05) between mean values were evaluated by one-way ANOVA and Duncan’s multiple range test.

## 4. Conclusions

The results showed an important effect of the analyzed genotypes on the composition of phytochemical compounds in the Saskatoon berry. Additionally, a positive correlation between antioxidant capacity and the content of bioactive compounds was found. The fruits of tested Saskatoon berry genotypes were found to be rich in phenolic compounds (average 5082.15 mg/100 g·dm), triterpenoids (average 78.93 mg/kg·dm), and carotenoids (average 520.10 mg/kg·dm), with high ABTS and FRAP capacity (average 22.43 and 17.5 mmol·Trolox/100 g·dm). It was also found that the fruits of the analyzed genotypes were a good source of pectins (average 1.0%), ash (average 0.63%), and sugars (average 14.36 g/100 g). Among the seven Saskatoon genotypes investigated, two new Polish clones (type S and type N) and two Canadian cultivars (“Honeywood” and “Smoky”) are characterized by the highest concentrations of phytochemical compounds and antioxidant properties. The obtained results and information will be useful for promoting the cultivation of these genotypes by commercial growers and amateurs in Poland. They will certainly attract the consumers’ interest in fruits of the Saskatoon berry due to their high contents of bioactive compounds and antioxidant capacity. In addition, they will provide a strong impetus for the food industry to develop attractive products based on the fruits of the Saskatoon berry that have potential health benefits. Valuable bioactive compounds, mainly anthocyanins, proanthocyanidins, triterpenoids, and carotenoids positive correlated with high antioxidant activity in Saskatoon berry, especially in the new clones type S and type N and cvs. “Honeywood” and “Smoky” could be applied to combat of free radicals.

## Figures and Tables

**Table 1:** Chemical composition of fruits in different Saskatoon berry genotypes analyzed in 2016.

Chemical Compounds	Honeywood	Martin	Pembina	Smoky	Clone No. 5/6	Clone Type N	Clone Type S
Dry substance (g/100 g) ^1^	22.12 ± 0.20c ^2^	20.8 ± 0.19cde	29.59 ± 0.27a	22.44 ± 0.20c	19.11 ± 0.17def	26.73 ± 0.24b	21.46 ± 0.19cd
Total acidity (g/100 g) ^1^	0.41 ± 0.00ab	0.57 ± 0.01ab	0.45 ± 0.00ab	0.44 ± 0.00ab	0.64 ± 0.01a	0.43 ± 0.00ab	0.37 ± 0.00ab
pH	3.78 ± 0.03ab	3.71 ± 0.03ab	3.02 ± 0.03ab	3.95 ± 0.04ab	3.99 ± 0.04a	3.65 ± 0.03ab	3.81±0.03ab
Pectins (%) ^1^	0.95 ± 0.01a	0.73 ± 0.01a	0.67 ± 0.01a	0.71 ± 0.01a	0.93 ± 0.01a	1.20 ± 0.01a	1.33 ± 0.01a
Ash (%) ^1^	1.11 ± 0.01a	0.84 ± 0.01a	0.71 ± 0.01a	0.91 ± 0.01a	0.61 ± 0.01a	1.03 ± 0.01a	1.14 ± 0.01a
Fructose (g/100g·dm) ^1^	6.83 ± 0.06ab	4.94 ± 0.04bc	5.08 ± 0.05abc	4.94 ± 0.04bc	3.14 ± 0.03d	6.07 ± 0.05ab	7.29 ± 0.07a
Sorbitol (g/100 g·dm) ^1^	2.25 ± 0.02a	2.41 ± 0.02a	1.84 ± 0.02a	3.37 ± 0.03a	1.80 ± 0.02a	2.19 ± 0.02a	2.49 ± 0.02a
Glucose (g/100 g·dm) ^1^	7.38 ± 0.07ab	2.50 ± 0.02c	6.18 ± 0.06b	7.16 ± 0.06ab	3.35 ± 0.03c	8.76 ± 0.08a	9.30 ± 0.08a
Sucrose (g/100 g·dm) ^1^	0.48 ± 0.00a	0.57 ± 0.01a	0.59 ± 0.01a	0.52 ± 0.00a	0.72 ± 0.01a	0.66 ± 0.01a	0.61 ± 0.01a
Σ sugar	16.95 ± 3.40b	10.42 ± 1.79d	13.69 ± 2.64c	15.99 ± 2.79b	9.02 ± 1.23d	17.68 ± 3.68ab	19.69 ± 4.05a

^1^ Values are means ± standard deviation, *n* = 3; ^2^ a–f Means ± SD followed by different letters within the same line represent significant differences (*p* < 0.05).

**Table 2:** The content of polyphenolic compounds (mg/100 g·dm) ^1^ of fruits in Saskatoon berry genotypes analyzed in 2016.

	Rt (min)	MS [H − M]^−^/[H − M]^+^	MS/MS Fragments (*m*/*z*)	Honeywood	Martin	Pembina	Smoky	Clone No. 5/6	Clone Type N	Clone Type S
*Anthocyanins*										
Cyanidin-3-*O*-galactoside ^3^	5.79	449.1685^+^	287.2555	1148.97 ± 4.02b	639.73 ± 2.24e	458.26 ± 1.60f	1107.16 ± 3.88c	461.06 ± 1.61fg	1013.49±3.55d	1219.07 ± 4.26a
Cyanidin-3-*O*-glucoside ^3^	6.27	449.1685^+^	287.2451	524.18 ± 1.83ab	291.86 ± 1.02d	209.07 ± 0.73f	505.11 ± 1.77b	210.34 ± 0.74e	462.37 ± 1.62c	556.16 ± 1.94a
Cyanidin-3-*O*-arabinoside ^3^	6.63	419.1101^+^	287.2451	196.24 ± 0.69b	109.26 ± 0.38e	78.27 ± 0.27f	189.10 ± 0.66c	78.75 ± 0.28f	173.10 ± 0.61cd	208.22 ± 0.72a
Cyanidin-3-*O*-xyloside ^3^	7.75	419.1722^+^	287.257	131.94 ± 0.46b	73.46 ± 0.26e	52.62 ± 0.18f	127.14 ± 0.44c	52.94 ± 0.19fg	116.38 ± 0.41d	139.99 ± 0.48a
**Σ Anthocyanins**				**2001.32 ± 304.42b ^2^**	**1114.31 ± 363.17e**	**798.22 ± 286.46g**	**1928.50 ± 418.90c**	**803.10 ± 248.58f**	**1765.32 ± 411.75d**	**2123.43 ± 442.23a**
*Phenolic acid*										
Protocatechuic acid ^4^	3.22	153.3815	109.6529	3.75±0.03ab	2.13±0.02bc	2.25±0.02bc	5.37±0.05a	1.69±0.02bc	2.99 ± 0.03bc	2.29 ± 0.02bc
Neochlorogenic acid ^3^	3.53	353.236	191.3855	181.02±1.63b	76.77±0.69d	52.50±0.47e	183.85±1.65a	175.76±1.58c	177.18 ± 1.59c	182.42 ± 1.64ab
p-hydroxybenzoic acid ^4^	4.00	137.4283	93.4301	52.06 ± 0.47a	13.96 ± 0.13e	33.43 ± 0.30c	47.20 ± 0.42b	6.27 ± 0.06f	53.73 ± 0.48a	30.38 ± 0.27d
Chlorogenic acid ^3^	4.74	353.9997	191.0571	770.00 ± 7.04b	384.55 ± 3.53f	140.01 ± 1.38g	655.63 ± 5.98d	501.27 ± 4.61e	670.00 ± 6.11c	872.10 ± 7.96a
Galic acid ^4^	4.78	169.3998	125.0691	11.95 ± 0.09c	8.21 ± 0.05g	13.19 ± 0.10a	9.31 ± 0.07e	10.87 ± 0.08d	9.03 ± 0.04ef	12.45 ± 0.05b
Cryptochlorogenic acid ^3^	5.05	353.236	191.3855	85.42 ± 0.77b	39.19 ± 0.35d	45.50 ± 0.41c	90.85 ± 0.82a	32.98 ± 0.30e	39.62 ± 0.36d	87.16 ± 078b
4-caffeoylquinic acid ^3^	6.10	353.1907	173.4518	47.87 ± 0.17	21.00 ± 0.07	27.53 ± 0.10	49.07 ± 0.17	35.31 ± 0.12	33.89 ± 0.12	15.53 ± 0.05
Caffeic acid glucoside ^4^	7.53	341.2219	179.3838	40.09 ± 0.15	17.59 ± 0.06	23.05 ± 0.08	41.09 ± 0.14	29.57 ± 0.10	28.38 ± 0.10	13.00 ± 0.04
Dicaffeic acid ^3^	8.34	353.9997	191.0996	2.96 ± 0.01	1.30 ± 0.00	1.70 ± 0.01	3.03 ± 0.01	2.18 ± 0.03	2.10 ± 0.01	0.96 ± 0.00
**Σ Phenolic acids**				**1195.12 ± 291.36b**	**564.70 ± 148.55f**	**339.15 ± 50.95g**	**1085.40 ± 244.45c**	**795.91 ± 196.59e**	**1016.92 ± 256.41d**	**1216.29 ± 340.16a**
*Flavonols*										
Kampferol-3-galactoside ^3^	6.03	447.2142	285.2469	44.92 ± 0.40a	19.12 ± 0.18c	18.62 ± 0.17c	45.54 ± 0.41a	19.35 ± 0.20c	44.59 ± 0.40a	41.04 ± 0.37b
Quercetin-3-*O*-arabinoglucoside ^4^	8.74	595.1489	301.2311	28.42 ± 0.10	14.78 ± 0.05	10.33 ± 0.04	25.98 ± 0.09	16.98 ± 0.06	23.91 ± 0.08	30.32 ± 0.10
Quercetin-3-*O*-rutinoside ^3^	9.15	609.1181	301.2352	39.54 ± 0.14	20.56 ± 0.07	14.37 ± 0.05	36.14 ± 0.13	23.62 ± 0.08	33.26 ± 0.12	42.18 ± 0.15
Quercetin-3-*O*-galactoside ^3^	9.36	463.1486	301.2321	209.57 ± 0.73	109.00 ± 0.38	76.16 ± 0.27	191.57 ± 0.67	125.22 ± 0.44	176.29 ± 0.62	223.58 ± 0.78
Quercetin-3-*O*-glucoside ^†^	9.61	463.1398	301.2286	21.66 ± 0.08	11.26 ± 0.04	7.87 ± 0.03	19.80 ± 0.07	12.94 ± 0.05	18.22 ± 0.06	23.10 ± 0.08
Quercetin-3-*O*-rabinobioside ^4^	9.82	609.9368	301.2037	14.78 ± 0.05	7.69 ± 0.03	5.37 ± 0.02	13.51 ± 0.05	8.83 ± 0.03	12.43 ± 0.04	15.77 ± 0.05
Quercetin-3-*O*-arabinoside ^3^	9.89	433.0698	301.0972	8.35 ± 0.03	4.34 ± 0.04	3.03 ± 0.01	7.63 ± 0.03	4.99 ± 0.02	7.02 ± 0.02	8.91 ± 0.03
Quercetin-3-*O*-xyloside ^3^	10.01	433.1374	301.225	7.16 ± 0.03	3.73 ± 0.01	2.60 ± 0.01	6.55 ± 0.02	4.28 ± 0.02	6.03 ± 0.02	7.64 ± 0.02
Quercetin-deoxyhexo-hexoside ^3^	10.29	609.108	301.257	7.85 ± 0.03	4.08 ± 0.01	2.85 ± 0.01	7.18 ± 0.03	4.69 ± 0.01	6.61 ± 0.02	8.38 ± 0.02
**Σ Flavonols**				**382.25 ± 206.77b**	**194.57 ± 110.54f**	**141.20 ± 73.51g**	**353.89 ± 185.83c**	**220.90 ± 128.83e**	**328.35 ± 169.12d**	**400.91 ± 225.45a**
*Flavan-3-ols*										
A-type procyanidin dimer ^3^	4.34	575.168	289.2398	22.81 ± 0.08	31.07 ± 0.11	46.41 ± 0.16	44.91 ± 0.16	21.46 ± 0.08	25.26 ± 0.09	24.85 ± 0.08
(+)-Catechin ^3^	5.48	289.111		18.70 ± 0.07	25.47 ± 0.09	38.05 ± 0.13	36.82 ± 0.13	17.59 ± 0.06	20.71 ± 0.07	20.37 ± 0.07
B-type procyjanidyn dimer ^3^	5.55	577.2162	407.1907/289.0727	2.75 ± 0.01	3.75 ± 0.01	5.60 ± 0.02	5.42 ± 0.02	2.59 ± 0.01	3.05 ± 0.01	3.00 ± 0.01
(−)-Epicatechin ^3^	6.57	289.2816		82.13 ± 0.29	111.84 ± 0.39	167.08 ± 0.59	161.67 ± 0.51	77.25 ± 0.27	90.94 ± 0.32	89.45 ± 0.32
B-type procyjanidyn trimer ^4^	7.22	865.0563	577.1276/287.3700	19.51 ± 0.07	26.57 ± 0.09	39.69 ± 0.14	38.40 ± 0.13	18.35 ± 0.06	21.60 ± 0.08	21.25 ± 0.07
B-type procyjanidyn tetramer ^4^	7.53	1153.077	865.1967/577.1158/287.2398	36.15 ± 0.13	49.23 ± 0.17	73.5 ± 0.26	71.16 ± 0.20	34.00 ± 0.12	40.02 ± 0.14	39.37 ± 0.13
A-type procyanidin trimer ^4^	7.60	863.168	575.1267/289.2467	36.46 ± 0.14	49.65 ± 0.10	74.18 ± 0.21	71.77 ± 0.15	34.30 ± 0.10	40.37 ± 0.10	39.71 ± 0.14
Polymeric procyanidin				2563.72 ± 23.07a	1602.79 ± 14.43e	1269.23 ± 11.42f	2259.02 ± 20.33d	1189.76 ± 10.71g	2480.65 ± 22.33b	2411.73 ± 23.69c
**Σ Flavan-3-ols**				**2782.24 ± 1658.31a**	**1900.36 ± 922.93e**	**1713.78 ± 583.14f**	**2689.17 ± 1293.21d**	**1395.30 ± 695.95g**	**2722.60 ± 1583.00b**	**2649.73 ± 1537.19c**
**Σ Polyphenolic compounds**				**6360.93 ± 57.25b**	**3773.94 ± 52.96e**	**2992.35 ± 26.93g**	**6056.96 ± 54.51c**	**3215.21 ± 28.94f**	**5833.20 ± 52.50d**	**6390.36 ± 57.51a**

^1^ Values are means ± standard deviation, *n* = 3; ^2^ a–g Means ± SD followed by different letters within the same line represent significant differences (*p* < 0.05); ^3^ Identification confirmed by commercial standards; ^4^ Identification by comparison of MS data with the literature and their identification is tentative.

**Table 3:** The fruit content of carotenoid, chlorophyll, and triterpenoid compounds (mg/kg of dm) ^1^ in seven Saskatoon genotypes analyzed in 2016.

Chemical Compounds	Rt (min)	MS [H − M]^−^/[H − M]^+^	MS/MS Fragments (*m*/*z*)	Honeywood	Martin	Pembina	Smoky	Clone No. 5/6	Clone Type N	Clone Type S
*Carotenoids*										
Zeaxanthin	4.66	601.0521		2.19 ± 0.02c ^2^	3.94 ± 0.04abc	3.01 ± 0.03bc	4.72 ± 0.04ab	2.83 ± 0.03bc	4.93 ± 0.04ab	5.32 ± 0.05a
all-*trans*-lutein	5.11	568.0021	551.1608/553.1281	165.60 ± 1.53b	153.16 ± 1.42d	146.96 ± 1.36e	164.70 ± 1.52b	141.78 ± 1.31f	162.49 ± 1.50c	168.99 ± 1.56a
13-*cis*-lutein	5.21	568.0261	551.1872/553.1023	4.65 ± 0.09c	4.30 ± 0.01e	4.13 ± 0.05g	4.62 ± 0.02d	3.98 ± 0.02f	4.56 ± 0.01b	4.75 ± 0.03a
β-carotene	8.72	537.1452	445.0571	344.23 ± 3.10c	336.71 ± 3.03e	324.52 ± 2.92g	340.32 ± 3.06d	330.98 ± 2.98f	380.21 ± 3.42b	382.51 ± 3.44a
Σ Carotenoids				516.67 ± 171.03c	498.11 ± 166.55e	478.62 ± 160.92g	514.36 ± 167.81d	479.57 ± 164.53f	552.19 ± 188.22b	561.57 ± 188.95a
*Chlorophylls*										
Chlorophyll b	7.30	907.1181	629.5560	39.81 ± 0.36e	27.76 ± 0.25f	56.12 ± 0.51a	23.04 ± 0.21g	54.12 ± 0.49ab	46.23 ± 0.42c	42.01 ± 0.38d
Pheophytin a	8.30	871.1362	593.2734/533.1276	47.12 ± 0.42d	52.34 ± 0.47b	49.46 ± 0.45c	48.16 ± 0.43cd	61.03 ± 0.55a	48.37 ± 0.44cd	51.98 ± 0.47b
Σ Chlorophylls				86.93 ± 5.17d	80.10 ± 17.38e	105.58 ± 4.71b	71.20 ± 17.76f	115.15 ± 4.89a	94.6 ± 1.51c	93.99 ± 7.05c
*Triterpenoids*										
Betulinic acid	6.89	455.3452^−^		11.62 ± 0.10a	7.45 ± 0.07cde	6.92 ± 0.06de	9.09 ± 0.08bcd	6.67 ± 0.06e	9.31 ± 0.08bc	10.84 ± 0.10ab
Oleanolic acid	7.58	455.3496^−^		71.03 ± 0.64c	57.83 ± 0.52f	56.02 ± 0.50f	64.92 ± 0.58d	62.94 ± 0.57de	73.14 ± 0.66b	75.38 ± 0.68a
Ursolic acid	8.19	455.3365^−^		6.19 ± 0.06a	3.68 ± 0.03bcd	3.61 ± 0.03bcd	3.95 ± 0.04abcd	2.60 ± 0.02cd	4.71 ± 0.04abc	5.09 ± 0.05ab
Σ Triterpenoids				88.84 ± 35.97b	68.96 ± 30.23e	66.55 ± 29.35f	77.96 ± 33.82c	72.21 ± 33.72d	87.16 ± 38.25b	91.31 ± 39.03a

^1^ Values are means ± standard deviation, *n* = 3; ^2^ a–g Means ± SD followed by different letters within the same line represent significant differences (*p* < 0.05).

**Table 4:** Correlation between antioxidant activity and bioactive compounds.

Polyphenolic Compounds	The Ability to Reduce Free Radical (ABTS)	Ferric Reducing Ability of Plasma (FRAP)
Betulinic acid	0.848	0.824
Oleanolic acid	0.840	0.827
Ursolic acid	0.713	0.452
Σ Triterpenoids	0.884	0.860
Σ Anthocyanins	0.932	0.915
Σ Phenolic acids	0.825	0.793
Σ Flavonols	0.882	0.857
Σ Flavan-3-ols	−0.543	−0.551
Polymeric procyanidin	0.957	0.935
Σ Phenolic compounds	0.925	0.900
Chlorophyll	−0.573	−0.563
Β-caroten	0.809	0.841
Zeaxanthin	0.496	0.599
Luteina	0.943	0.939
Σ Carotenoid compounds	0.905	0.928
